# Quality control in clinical raster-scan optoacoustic mesoscopy

**DOI:** 10.1016/j.pacs.2023.100582

**Published:** 2023-12-22

**Authors:** Hailong He, Chiara Fischer, Ulf Darsow, Juan Aguirre, Vasilis Ntziachristos

**Affiliations:** a Institute of Biological and Medical Imaging, Helmholtz Zentrum München, Neuherberg, Germany; b Chair of Biological Imaging at the Central Institute for Translational Cancer Research (TranslaTUM), School of Medicine, Technical University of Munich, Munich, Germany; c Department of Dermatology and Allergy, Technical University of Munich, Munich, Germany; d Departamento de Tecnología Electrónica y de las Comunicaciones, Universidad Autónoma de Madrid, Madrid, Spain; e Instituto de Investigación Sanitaria de la Fundación Jimenez Diaz, Madrid, Spain

**Keywords:** Photoacoustic, Skin imaging, Quality evaluation, Optoacoustic mesoscopy, Motion correction

## Abstract

Optoacoustic (photoacoustic) mesoscopy bridges the gap between optoacoustic microscopy and macroscopy and enables high-resolution visualization deeper than optical microscopy. Nevertheless, as images may be affected by motion and noise, it is critical to develop methodologies that offer standardization and quality control to ensure that high-quality datasets are reproducibly obtained from patient scans. Such development is particularly important for ensuring reliability in applying machine learning methods or for reliably measuring disease biomarkers. We propose herein a quality control scheme to assess the quality of data collected. A reference scan of a suture phantom is performed to characterize the system noise level before each raster-scan optoacoustic mesoscopy (RSOM) measurement. Using the recorded RSOM data, we develop a method that estimates the amount of motion in the raw data. These motion metrics are employed to classify the quality of raw data collected and derive a quality assessment index (*QASIN*) for each raw measurement. Using simulations, we propose a selection criterion of images with sufficient *QASIN*, leading to the compilation of RSOM datasets with consistent quality. Using 160 RSOM measurements from healthy volunteers, we show that RSOM images that were selected using *QASIN* were of higher quality and fidelity compared to non-selected images. We discuss how this quality control scheme can enable the standardization of RSOM images for clinical and biomedical applications.

## 1 Introduction

Raster-scan optoacoustic mesoscopy (RSOM) yields high-quality and high-fidelity performance by utilizing broadband ultrasound transducers in the tens to hundreds of MHz, achieving resolutions in the tens of microns or better through millimeters of tissues [1], [2], [3], [4], [5], [6], [7], [8]. Despite demonstrating new imaging ability, RSOM image quality is sensitive to motion, fluctuations of laser intensity and electrical noise [9], [10], [11]. Light attenuation in tissues and the effects of skin tone on the optoacoustic signal may also affect the signal collected and image quality [12], [13]. As this technology is increasingly considered for clinical handheld applications, it is critical to pursue strategies that assess the quality of data collected and ensure consistency in measurements. Such assessment could be used for issuing warnings during the acquisition process, for quality control purposes in clinical studies, or for generating datasets of consistent quality for training of analysis algorithms [10], [11], [14].

The effect of motion on optoacoustic data has been previously studied and can be divided into two groups: periodic displacements due to tissue physiology, in particular arterial pulsation and heartbeat, and random muscular movement during acquisition [10]. During a given measurement, skin displacement normal to the detector surface (vertical displacement) in the tens to hundreds of micrometers can be observed [10]. We have reported two motion correction algorithms that address the effects of motion in RSOM systems [9], [10]. Schwarz et al. first introduced a motion correction algorithm that relied on segmentation of the skin’s melanin layer [9]. Aguirre et al. further studied the origin and magnitude of vertical displacements of skin, and proposed an automated motion correction algorithm based on cross-correlation functions between raw data (A-lines or B-planes) [10]. These studies have shown that motion can significantly affect image quality and that suggested motion correction algorithms can offer marked improvements [9], [10], [14]. Nevertheless, the overall improvement afforded by motion correction algorithms varies depending on the number of motion effects present in the data [9], [10], [15], [16], [17], [18] and the overall signal-to-noise ratio (*SNR*).

In this work we aimed to develop a scheme that could assess the quality and uniformity of RSOM datasets and provide an estimate of the resulting image fidelity. An implicit goal was to suggest methodology that reads all necessary information directly from the raw data so that it can be ubiquitously applied to datasets obtained from different experimental systems, without the need of additional measurements requiring specialized hardware. While such analysis could also be performed directly at the image space, we consider quality extraction from analyses of raw data to develop a tool that could be used even during the acquisition process and that is independent of the image-reconstruction-algorithm. We hypothesized that raw data contains sufficient information to extract parameters that describe the quality of the acquisition, and that this information could be summarized in a quality assessment index (*QASIN*) for the dataset. To minimize the noise variations, we characterized the RSOM system noise level by measuring a common phantom, ensuring the system performed consistently for every scan. Then we extracted motion variations found in the raw data to suggest a *QASIN*. Subsequently, using simulations, we evaluated the relationship of the *QASIN* to image quality and validated the performance of this quality index on RSOM data obtained from 160 measurements on volunteers. We show that while motion-corrected images for *QASIN* values below a certain threshold result in marked image quality improvements, the same algorithms do not provide effective correction of data for values above the threshold value. We discuss how application of *QASIN* can help ensure high-fidelity data collection and improve the reliability of clinical measurements.

## 2 Methods

### 2.1 RSOM system

**Fig. 1:**
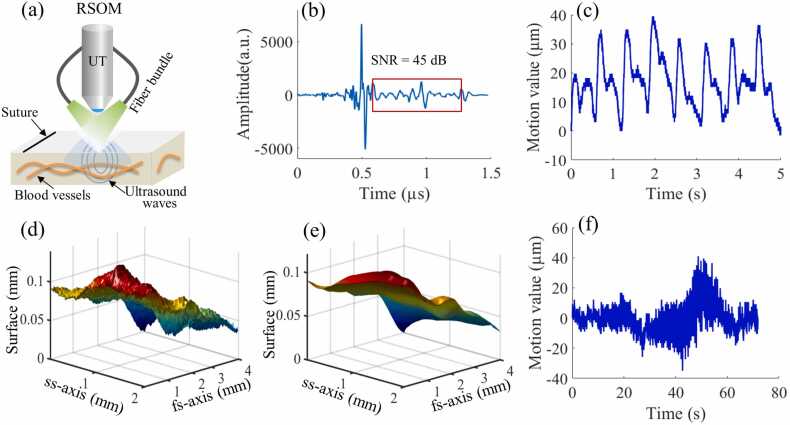
The scanning process of clinical raster-scan optoacoustic mesoscopy (RSOM). (a) Schematic of the RSOM scanning head, containing a 50 MHz ultrasound transducer (UT) and two fiber bundle. The head is positioned over the skin, where the region of interest (ROI) is covered with a transparent plastic foil. The reference suture for *SNR* characterization is illustrated as a thick black line. **(b)** A representative optoacoustic signal of the reference suture acquired at the optimal distance between the suture and the RSOM head. The *SNR* value is calculated as the ratio between peak single intensity and the standard deviation of noise background marked by the red rectangle. **(c)** Motion graph of point measurements acquired at the wrist pulse area from a healthy volunteer. **(d, e)** Skin surfaces extracted from RSOM data: the disrupted surface *S*_D_ (d) and the smoothed surface *S*_C_ (e). *S*_D_ and *S*_C_ are two dimensions (m×n) with the same size of the recorded RSOM data, m is the scanning position number in fast scan (fs) axis and n represents the scanning position number in slow scan (ss) axis. **(f)** Motion graph calculated by subtracting the disrupted surface (e) from the smoothed surface (f).

The present study used an in-house RSOM system featuring a transducer with broad bandwidth (10–120 MHz) and central frequency of 50 MHz. Illumination was provided by a pulsed laser at a wavelength of 532 nm with repetition rate of 500 Hz, yielding an optical fluence (3.75 µJ/mm2) that is under the safety limit according to the American National Standards for Safe Use of Lasers in humans [1]. An optically and acoustically transparent plastic membrane (light grey rectangle, Fig. 1a) was affixed to the patient's skin at the region of interest (ROI) using surgical tape. The scanning head containing the fiber bundle and transducer was brought close to the membrane to position the focal point of the ultrasound detector slightly above the skin surface and thereby maximize detection sensitivity [1], [10]. Two mechanical stages (PI, Germany) were used to move the RSOM head. Two mechanical stages (PI, Germany) were used to move the RSOM head. The scanning head contained water as a coupling medium. Detailed information of our RSOM imaging setup has been described in our previous work [1], [19].

### 2.2 SNR reference test

The *SNR* of clinical RSOM data is primarily affected by light fluence attenuation inside tissue, the strength and intensity variations of the illumination source and sources of electrical noise [13]. In a previous study, we investigated the effect of skin phototype on the SNR of optoacoustic signals collected from the human dermis and suggested that compensating for signal intensity variations due to melanin content could improve the performance of quantitative analysis [12].

Herein, we established a reference measurement by attaching a black surgical polyamide suture (Braun, Germany, 100 µm in diameter) to the transparent membrane used for coupling the RSOM system to tissue (Fig. 1). Measurements over the suture provide a consistent measurement which reports on system reproducibility. The suture was scanned with 266 × 1 points (step size of 15 µm) in each RSOM scan, over a period of 0.2 s. The distance between the RSOM scanning head and skin was chosen based on the maximum *SNR* measured from the suture. The maximum *SNR* value is taken as the reference value of the suture *SNR* test (*R*_SNR_), which characterizes the functional noise level of the RSOM system. For every RSOM scan, the *SNR* of suture signals is first calculated and the full RSOM scan begins only when the *SNR* of the suture signal is above *R*_SNR_. When the *SNR* is below *R*_SNR_, system components like laser energy, or the coupling between the device and the tissue should be optimized before starting a full RSOM scan.

### 2.3 Quantification of motion

The noise of the RSOM system is calibrated by the suture reference test, achieving a standardized system noise level. Following this, we aimed to standardize the quantification of the amount of motion in the recorded RSOM data. As we have previously reported [9], the surface extraction-based motion correction algorithm is implemented by observing disruptions of the strong optoacoustic signals generated by the vertical movement of the melanin layer at the skin surface. A corresponding three-dimensional map of a skin surface that is disrupted by motion (*S*_D_) can be generated by aligning the maximum signal intensity of each scanning position from the RSOM scan (correlating to the melanin layer, see Fig. 1d). The disrupted skin surface can then be smoothed to obtain an artificial continuous surface (*S*_C_, see Fig. 1e). The differences between the two surfaces are assumed to be a result of the vertical motion (*M*) of the skin with respect to the detector:

(1) *M* = *S*_D_ – *S*_C_

The three-dimensional maps of motion were transformed into a two-dimensional graph (see Fig. 1f) along the scanning time points for visualization. The standard deviation (*M*_std_) and maximum motion (*M*_max_) values of the motion graph were computed to characterize the overall motion of the skin. To quantify the motion levels of recorded RSOM data, we formulated the following quality assessment index of motion (*Q*_motion_):

(2) *Q*_motion_ = *M*_std_ + β*M_max_

The threshold of *Q*_motion_ is determined by thresholds of *M*_std_ and *M*_max_ (*T*_std_ and *T*_max_). As a weighting value to balance the contributions between *M*_std_ and *M*_max_. *β* is defined as *T*_std_ /*T*_max_, which is affected by the maximum motion that our motion correction algorithm can handle, and is explained in detail in the following section.

### 2.4 Simulations to determine thresholds of Q_motion_

**Fig. 2:**
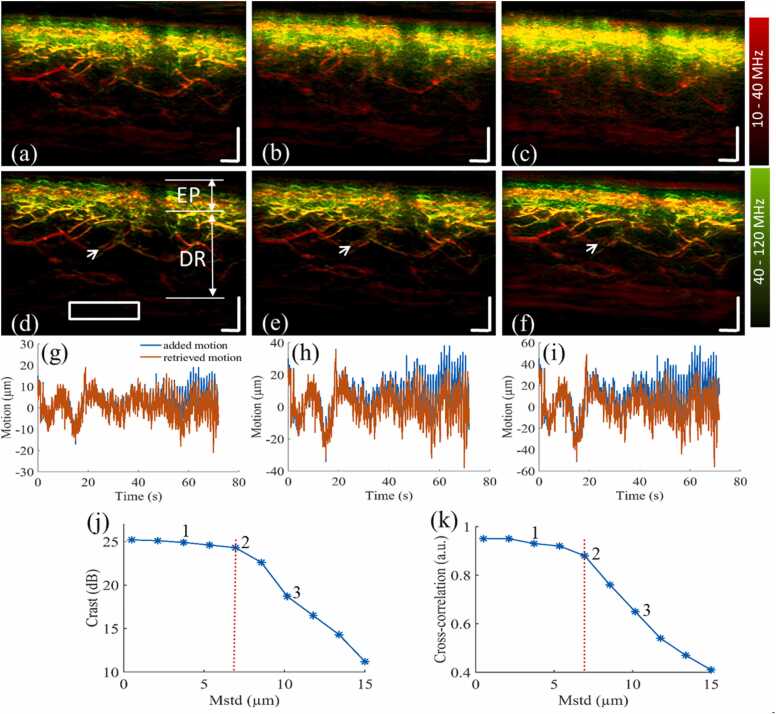
Simulations to determine threshold values of *Q*_motion_ for classifying raster-scan optoacoustic mesoscopy (RSOM) images as low- or high-quality. The corrupted RSOM datasets are formed by adding artificial motion graphs with different variations to motionless RSOM raw data. **(a-c)** Three reconstructed maximum intensity projection (MIP) images after adding motion graphs (corresponding to labels 1–3 in j and k) to the RSOM raw data without motion correction. **(d-f)** Corresponding reconstructed MIP images after adding motion graphs to the RSOM raw data with motion correction. The images are color-coded to represent the two reconstructed frequency bands (red: larger structures in the bandwidth of 10–40 MHz; green: smaller structures in the bandwidth of 40–120 MHz). The skin epidermis (EP) and dermis (DR) layers are indicated in (d). **(g-i)** Comparisons between the added motion graphs (blue) and the corresponding retrieved motion graphs (red). **(j)** Relationship between changes of the contrast-to-noise ratio (*CNR*) and *M*_std_ of the added motion graphs. **(k)** Cross-correlation values between the added and retrieved motion graphs. Labels 1–3 in (j) and (k) indicate the *M*_std_ values of images (a)-(c).The red dashed lines indicate the determined value of *T*_std_. Scale bar 500 µm.

RSOM data was recorded with consistent system noise level characterized by the SNR of the suture reference scan. The RSOM data and corresponding image quality were further assessed based on whether the amount of motion contaminating the data was likely to be less or greater than what our motion correction algorithm could handle. In this section, we investigated how motion correction algorithms performed for different amplitudes of motion and applied simulation studies to determine *T*_std_ and *T*_max_. A base motion graph [blue line in Fig. 2g] was extracted from RSOM data acquired over a skin region of 4 × 2 mm in the lower arm of a healthy volunteer, where pulse, breathing and random motions are mixed, and was then treated as a complex vertical motion pattern. Ten artificial motion graphs were generated by multiplying weighting values (0.1 to 3 with step size of 0.3) of the base motion graph, achieving standard deviations from 0.5 µm to 15 µm indicated by the blue stars in Fig. 2j and corresponding maximum motions from 4 to 120 µm. Then, the artificial motion graphs were added to a motion-corrected RSOM image shown in Fig. 2d, obtaining a sequence of motion-corrupted RSOM datasets. The motion correction algorithm developed by our previous study [9] was then applied to correct the motion-corrupted RSOM data. The differences between the added motions and the retrieved motions from the motion-corrupted data are characterized by cross-correlation *C(n)*:

(3) $$C(n)=M_{a}^{n}*M_{r}^{n}$$

Where *n* represents the number of the added motion graphs. $M_{a}^{n}$ is the *n*th added motion graph and $M_{r}^{n}$ corresponds to the *n*th retrieved motion graph. * is the cross-correlation operator.

The contrast to noise ratio (*CNR*) of the motion corrected images was calculated to quantify the performance of the motion correction algorithm on the ten motion-corrupted RSOM datasets. We defined *CNR* as:

(4) $$\mathrm{CNR}=I_{p}/S_{b}$$

where $I_{p}$ represents the peak intensity of RSOM features inside the reconstructed image. $S_{b}$ refers to the standard deviation of a background region in the reconstructed image. The values of *T*_std_ and *T*_max_ were determined based on the decrement of the *CNR* values, where it dropped by 1 dB. According to Eq. 2, the threshold of *Q*_motion_ (*TQ*_motion_) equals: *T*_std_ + β * T_max_.

### 2.5 RSOM measurements

In order to assess physiological motions inside the scanned area, the RSOM head was fixed for 5 s as a point measurement, and the vertical displacement of the point measurements were calculated based on cross-correlation methods from the collected optoacoustic A-line signals [10]. A point measurement at the wrist pulse area of a healthy volunteer was recorded as depicted in Fig. 1c. An RSOM scan of the lower arm from the healthy volunteer was also recorded for the simulations to determine *T*_std_ and *T*_max_, and corresponding *TQ*_motion_ values as shown in Fig. 2. To validate the quality control method, we acquired 160 RSOM measurements at the lower extremities (pretibial area) of 80 volunteers (two measurements per volunteer) to evaluate the *TQ*_motion_. All measurements were approved by the ethics committee of the Technical University of Munich. For all RSOM measurements, 266 × 135 points were scanned in an area of 4 × 2 mm. The scanning time was 70 s. All volunteers were provided with written informed consent. Procedures were conducted in accordance with institutional and international guidelines.

## 3 Results

### 3.1 The SNR reference scan and the quantification of motion in RSOM data

The schematic illustration of the RSOM head is shown in Fig. 1a, where the black line indicates the position of the suture. The maximum intensity of the suture signals (Fig. 1b) was obtained at the position where the suture was located at the focal point of the ultrasound transducer, generating a maximal *SNR* value of 45 ± 0.3 dB (five repeated measurements), which is defined as *T*_SNR_. Fig. 1c shows motion graphs of point measurements at the wrist pulse area (30 ± 5 µm), where periodical motions introduced by arterial pulsation were clearly resolved. The detected skin surfaces before and after motion correction of the RSOM data acquired from the healthy volunteer at the lower arm are shown in Figs. 1d and 1e, while the corresponding motion graph calculated based on Eq. 1 is depicted in Fig. 1f. The motion graph of the RSOM data contained mixed movements induced by arterial pulsation, wrist pulse and random muscular movement. Small displacements in the range of 10 ± 5 µm were observed in the first 30 s while large motions up to 35 ± 5 µm appeared after 40 s of scanning.

### 3.2 Threshold determination of Q_motion_ by simulation study

Simulations were performed to determine thresholds of *Q*_motion_ for classifying RSOM scans as low- or high-quality. The first row (Fig. 2a-c) shows the motion-corrupted RSOM images, while the corresponding motion-corrected images (Fig. 2d-f) are shown in the second row. The added motion graphs (blue lines) and the retrieved motion graphs (red lines) with different level of motion are depicted in Fig. 2g-i. Similarities between the ten added and retrieved motion graphs characterized by the cross-correlation values are shown in Fig. 2j. Labels (1, 2, 3) in Fig. 2j correspond to the added motion graphs from images in Fig. 2a-c respectively. We note that the similarities between the added and retrieved motion graphs were reduced with increments of *M*_std_ in the ten added motion graphs. The cross-correlation values dropped significantly from point 2 to point 3 in Fig. 2j, which correlates with the image quality distortion in the motion corrected images as shown in Fig. 2e and f. The *CNR* values of the ten motion corrected images calculated by the ratio between the peak image intensity marked by the white arrows in Fig. 2d-f and standard deviations of the background area inside the white rectangle (Fig. 2d) are shown in Fig. 2k, which show similar changes of the cross-correlation values. The *M*_std_ (7 µm) and corresponding *M*_max_ values (75 µm) marked by the red dash lines in Fig. 2(j) and (k) were selected as the *T*_max_ and *T*_std_, which was the turning point where the cross-correlation value and image contrast (reduced by 1 dB) both decreased significantly after point 2.

### 3.3 Quality assessment of RSOM datasets based on motion values

**Fig. 3:**
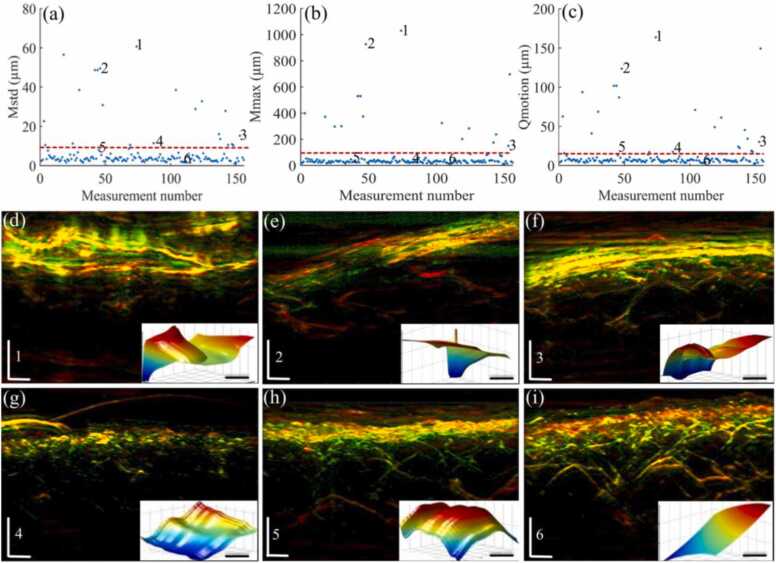
Quality assessment of 160 clinical raster-scan optoacoustic mesoscopy (RSOM) datasets based on *M*_std_, *M*_max_ and *Q*_motion_ values and corresponding thresholds. (a, b) *M*_std_ and *M*_max_ values of motion graphs computed from 160 RSOM datasets. (c) corresponding *Q*_motion_ values. Red dashed lines indicate the positions of *T*_std_, *T*_max_ and *TQ*_motion_ values. **(d-i)** Cross-sectional maximum intensity projection (MIP) RSOM images, correspond to labels 1–6 marked in (a)-(c), which show various image quality, scale bar: 500 µm. The insets show the smoothed surfaces$\;S_{C}$of RSOM data, scale bar: 1 mm.

We extracted motion values from a large clinical RSOM dataset according to the procedure outlined in Section 2. C and compared them to the threshold values determined in Section 2.D. Our goal was to demonstrate whether we could identify low-quality scans that could then be excluded to generate a dataset of uniformly high quality, which would facilitate quantitative analysis in large clinical studies. For this, we validated *Q*_motion_ on a dataset of 160 RSOM measurements from the lower legs of 80 volunteers (two measurements per volunteer). Fig. 3a,b plot the *M*_std_ and *M*_max_ values calculated from the motion graphs of 160 RSOM scans and corresponding *T*_std_ and *T*_max_ indicated by the red dashed lines. The *Q*_motion_ values calculated based on Eq. 2 is shown in Fig. 3c, where the red dashed line indicates the threshold *TQ*_motion_. It can be noted that 22 datasets were above the threshold *T*_std_ and 17 datasets were above the threshold *T*_max_. All datasets above *T*_std_ and *T*_max_ were identified by the *TQ*_motion_ value as shown in Fig. 3c. Fig. 3d-i show six representative images with *M*_std_, *M*_max_ and *Q*_motion_ values corresponding to labels (1−6) in Fig. 3a-c. Smoothed surface (*S*_c_) of each data are displayed in the insets of Fig. 3d-i. It can be seen that the *M*_std_ and *M*_max_ values correspond well with the variations of the smoothed surface (*S*_c_). The image quality improvement is correlated with the decrease observed in *M*_std_, *M*_max_ and *Q*_motion_ values. For example, Fig. 3d,e, corresponding to labels 1 and 2 with high *Q*_motion_ values, shows markedly low image quality where obvious outliers were seen in the smoothed surface. Labels 3 and 4 corresponding to the images shown in Fig. 3f,g present moderate quality with a smoother surface, which had *Q*_motion_ values close to the threshold lines. Fig. 3h,i corresponding to labels 5 and 6, depict higher image quality with both *Q*_motion_ values below the threshold values compared to Fig. 3d-g.

## 4 Discussion

In this work, we developed a quality assessment index (*QASIN*) to quantify and evaluate motion in order to keep data and image quality consistency among different RSOM scans. To do this, we defined the derivation of quantities for both the maximum (*M*_*max*_) and the standard deviation (*M*_*std*_) of the motion in a RSOM scan. We then introduced a method for determining a threshold for the maximum correctable motion per scan. These values were validated on 160 RSOM scans of the lower legs of volunteers, which showed that the motion artefacts in images with *M*_*max*_ and *M*_*std*_ above the determined thresholds could not be properly corrected, resulting in low image quality. We additionally applied an external standard to test the maximum *SNR* of the system prior to measuring, further improving the consistency of RSOM measurements. The proposed quality control method enables high-fidelity data collection and improves the reliability of quantification analysis for RSOM studies.

The *SNR* of the reference suture signal was first calculated and the full RSOM data was recorded only when the *SNR* was above the *T*_SNR_ value of 45 dB, which allowed us to minimize variations of system performance between scans from same people, scans from different people, or scans from different RSOM setups. The reference scan will be implemented as a calibration procedure of commercial RSOM setups in the future. With the reference test, we could correctly position and keep the same distance between the RSOM scanning head and tissue for all scans. The reference test can further help to check the laser energy fluctuations, and the coupling quality between the detector and tissue, since air bubbles or other incorrect coupling can easily degrade the signal quality. In order to monitor signal quality during the whole RSOM scan period, we plan to position a thin film made of low absorption material between the transducer and the skin surface, which will generate a continuous reference optoacoustic signal to calibrate the system performance. In the future, we can investigate the effect of skin phototype on SNR in the dermis by analyzing the SNR of skin surface signals recorded during fast line suture measurements.

We further reported the *Q*_motion_ that quantitatively classifies the recorded RSOM data quality based on the amount of motion contaminating the data. The threshold *T*_std_ and *T*_max_ values of *Q*_motion_ were determined based on the maximum motion that our motion correction algorithm could handle. As shown in Fig. 3, the *Q*_motion_ was evaluated on 160 clinical RSOM scans, showing good correlation with the reconstructed image quality. Previous motion correction methods have demonstrated significant improvements of image quality, but the correction improvements were not uniform at different RSOM scans. For example, serious motions up to 500 µm were seen in the motion surface of RSOM data shown in Fig. 3c-e, which may be caused by human jitter movements. Those motions with large variations posed challenges to the motion correction algorithm, resulting in inconsistent correction improvements. As reported in Fig. 3, 17 low quality datasets were determined by the *T*_std_ and *T*_max_ values simultaneously, while 6 more datasets were further selected based on *T*_std_ value. However, all low-quality datasets identified by the *T*_std_ and *T*_max_ values are above the threshold of *TQ*_motion_. Therefore, the *TQ*_motion_ value is determined as the motion *QASIN*. Selecting RSOM datasets with *Q*_motion_ smaller than *TQ*_motion_ allowed us to obtain consistent motion correction improvements.

The motion graphs of RSOM data and corresponding threshold values were calculated based on the surface motion correction algorithm developed by Schwarz et al.[9]. The skin surface was determined based on segmentation of the skin's melanin layer in the three-dimensional sinogram, from which the disruptions could be quantified and corrected. The algorithm developed by Schwarz et al. [9] may not work without sufficient melanin to generate a detectable optoacoustic signal. However, a cross-correlation based motion correction approach introduced by Aguirre et al. [10], which does not need anatomical segmentation, can be applied to calculate the motion graphs for our developed data quality assessment scheme. New simulation studies should be performed to investigate *T*_std_ and *T*_max_ when applying different motion correction algorithms. In addition, the threshold value *T*_std_ and *T*_max_ of the *Q*_motion_ was determined by simulations based on a specific motion pattern as shown in Fig. 3g. More complex motion patterns can be used in the simulation studies to further optimize the determination of *T*_std_ and *T*_max_. Our data quality control method is determined by the computation accuracy of motion graphs derived from the motion correction algorithms. As the tissue motion in human skin is very complex, signal analysis-based motion correction algorithms are limited to a certain motion level and correction errors are not uniform when handling different levels of motion. Besides extracting from the recorded data, the motion graphs of RSOM measurements can be obtained by using a laser distance meter that enables real-time tracking of skin movements, which can be integrated with the RSOM scanning head to allow real-time monitoring of motion during RSOM scanning. Moreover, rigid and non-rigid motion correction approaches can be combined to correct motion for future improvements [15], [17], [18].

The motion control method can select recorded RSOM data with similar motion levels, which can minimize motion effects in the final reconstructed image, resulting in uniform RSOM image quality. The QASIN is determined based on the motion value computed from the recorded RSOM raw data, which is independent of illumination wavelengths. Multi-wavelength RSOM requires the same quality in each wavelength scan. QASIN can select RSOM data of the same quality at each wavelength by excluding low quality scans with obviously high motion values, ensuring consistent quality of multi-wavelength RSOM data. The QASIN method can be applied to evaluate the data quality of other optoacoustic imaging systems, such as optoacoustic optical/acoustic resolution microscopy, based on the quantification of motion recorded during the scanning period. RSOM has shown great skin imaging performance, which existing techniques cannot achieve, enabling novel clinical applications such as precise psoriasis treatment monitoring [7], melanoma non-invasive detection [6] and investigation of diabetic skin microvasculature complications [8]. To further demonstrate the clinical potential of RSOM, large-scale clinical studies, with uniform image quality evaluated by a quality control scheme, are needed for the quantitative analysis of RSOM images.

In conclusion, we developed a quality control scheme to evaluate RSOM data quality. In this scheme, the *SNR* reference scan allows the maintenance of consistent system performance between different scans or different imaging setups. The quality of RSOM datasets is evaluated by the *Q*_motion_ values, and data with server motions beyond the threshold of the *Q*_motion_ are excluded, resulting in consistent motion correction performance. Overall, the quality control scheme enables clinical RSOM images with uniformly high quality, which promotes quantitative analysis of RSOM images for applications in biology and clinics.

## CRediT authorship contribution statement

**Darsow Ulf:** Investigation, Methodology, Writing – review & editing. **Aguirre Juan:** Conceptualization, Investigation, Methodology, Writing – review & editing. **He Hailong:** Conceptualization, Data curation, Formal analysis, Investigation, Methodology, Writing – original draft, Writing – review & editing. **Fischer Chiara:** Investigation, Methodology, Writing – review & editing. **Ntziachristos Vasilis:** Conceptualization, Funding acquisition, Investigation, Project administration, Supervision, Writing – original draft, Writing – review & editing.

## Declaration of Competing Interest

The authors declare the following financial interests/personal relationships which may be considered as potential competing interests: Vasilis Ntziachristos is a founder and equity owner of sThesis GmbH, iThera Medical GmbH, Spear UG and I3 Inc. All other authors declare that they have no known competing financial interests or personal relationships that influenced the work reported in this paper.

## Data Availability

Data will be made available on request.
